# Regulation of NEIL1 protein abundance by RAD9 is important for efficient base excision repair

**DOI:** 10.1093/nar/gkv327

**Published:** 2015-04-14

**Authors:** Sunil K. Panigrahi, Kevin M. Hopkins, Howard B. Lieberman

**Affiliations:** 1 Center for Radiological Research, College of Physicians and Surgeons, Columbia University Medical Center, New York, NY 10032, USA; 2 Department of Environmental Health Sciences, Mailman School of Public Health, Columbia University Medical Center, New York, NY 10032, USA

## Abstract

RAD9 participates in DNA damage-induced cell cycle checkpoints and DNA repair. As a member of the RAD9-HUS1-RAD1 (9-1-1) complex, it can sense DNA damage and recruit ATR to damage sites. RAD9 binding can enhance activities of members of different DNA repair pathways, including NEIL1 DNA glycosylase, which initiates base excision repair (BER) by removing damaged DNA bases. Moreover, RAD9 can act independently of 9-1-1 as a gene-specific transcription factor. Herein, we show that mouse *Rad9^−/−^* relative to *Rad9*^+/+^ embryonic stem (ES) cells have reduced levels of Neil1 protein. Also, human prostate cancer cells, DU145 and PC-3, knocked down for *RAD9* demonstrate reduced NEIL1 abundance relative to controls. We found that Rad9 is required for Neil1 protein stability in mouse ES cells, whereas it regulates *NEIL1* transcription in the human cells. RAD9 depletion enhances sensitivity to UV, gamma rays and menadione, but ectopic expression of *RAD9* or *NEIL1* restores resistance. Glycosylase/apurinic lyase activity was reduced in *Rad9^−/−^* mouse ES and *RAD9* knocked-down human prostate cancer whole cell extracts, relative to controls. Neil1 or Rad9 addition restored this incision activity. Thus, we demonstrate that RAD9 regulates BER by controlling NEIL1 protein levels, albeit by different mechanisms in human prostate cancer versus mouse ES cells.

## INTRODUCTION

The genomic integrity of cells is always challenged because of exposure to DNA damaging agents, either from exogenous sources, including radiations and chemicals, or endogenous toxic metabolites such as reactive oxygen species and free radicals (1). Complex DNA damage response (DDR) pathways maintain genomic stability. The DDR network is comprised of a set of tightly coordinated processes that include detection of DNA damage, a signaling cascade, recruitment of repair factors to the DNA damage site and repair (2). To ensure genomic stability, DDR proteins recognize many types of aberrant DNA structural alterations, including nicks, gaps, base mismatches, single as well as double strand breaks, and also aberrations due to stalled DNA replication forks. DNA damage-induced cell cycle checkpoints promote genome stability through transient delays in cell cycle progression that allow cells to repair DNA lesions before entering critical phases of the cell cycle. Proteins involved in this pathway are regulated through a wide range of processes, including transcriptional and post-transcriptional control (3), protein–protein interactions (4) and subcellular localization (5). Aberration in these processes can lead to cancer, immunodeficiency and neurological disorders (6,7).

Base excision repair (BER) is an evolutionarily conserved process that mends a wide range of nucleotide alterations, including abasic sites (8). BER is initiated by a DNA glycosylase, which removes damaged nitrogenous bases by catalyzing hydrolysis of the N-glycosidic bond (9,10). DNA glycosylases are small monomeric proteins that can be classified into two groups based on functionality: (i) Mono-functional, which recognizes lesions and recruits an AP-endonuclease (APE1) to create a nick 5′ to the baseless site, and later removes the baseless sugar residue and (ii) Bi-functional, which recognizes and removes lesions by intrinsic lyase activity. However, based on substrate specificity and structural motifs, DNA glycosylases can also be classified into two families: (i) Fpg/Nei and (ii) Nth. In mammals, three Fpg/Nei family members have been identified, namely NEIL1, NEIL2 and NEIL3 (11). NEIL1 and NEIL2 are well characterized biochemically. Both are bi-functional enzymes that incise damaged DNA by β, δ-elimination, and also are involved in an APE1-independent BER pathway (12). NEIL3 is a mono-functional enzyme and has only β-elimination incision activity (13). NEIL1 prefers duplex DNA structures more than single-stranded DNA or forks as substrate, whereas both NEIL2 and NEIL3 have the opposite preferences (11). NEIL1 interacts with many DNA replication proteins and is involved in removal of DNA lesions during replication (12,14,15). However, NEIL2 participates in transcription-coupled repair due to its preference for single-stranded DNA and interaction with RNA polymerase II, along with many transcription factors (16). As compared to NEIL2 and NEIL3, NEIL1 recognizes a wide variety of lesions and is responsible for repairing a diverse set of DNA modifications, including base oxidation and apurination.

The RAD9-HUS1-RAD1 (9-1-1) heterotrimeric complex is loaded onto chromatin by the RAD17-Replication factor C clamp loader when DNA damage is incurred (17). RAD9 is a multi**-**functional protein that interacts with several DNA repair proteins either as part of 9-1-1 or independently (18). Unlike RAD1 or HUS1, RAD9 can also function as a transcriptional activator for specific downstream target genes (19). Furthermore, RAD9 participates in most DNA repair mechanisms, including BER (20), nucleotide excision repair (21), mismatch repair (22) and homologous recombination repair (23). Broadly, RAD9 can be divided into two functional regions. The N-terminal region contains a pro-apoptotic BH3-like domain, an exonuclease domain and two proliferating cell nuclear antigen (PCNA)-like domains, involved in 9-1-1 formation. In addition, it also contains several sites important for the interaction with other proteins, such as CAD (carbamoyl phosphate synthetase/aspartate transcarbomylase/dihydroorotase), TRP2 (tetratricopeptide repeat protein 2), MLH1 (MutL homolog 1) and TLK1 (tousled like kinase 1) (24). The C-terminal region is intrinsically disordered and contains 10 phosphorylation sites (25,26), a nuclear localization signal (27), and binding sites for other proteins involved in the DDR, such as RPA (28) and TopBP1 (25). Furthermore, it has a binding site for androgen receptor (AR) and acts as a co-regulator to suppress androgen-AR transactivation function in prostrate cancer cells. For BER, the 9-1-1 complex and also RAD9 independently interact with NEIL1 protein (29), resulting in the stimulation of NEIL1 activity (29).

In this study, we demonstrate another role for RAD9 in BER. We show that RAD9 regulates the abundance of NEIL1 protein. In human prostate cancer cell lines, DU145 and PC-3, RAD9 does this by activating *NEIL1* transcription. However, in mouse ES cells, Rad9 stabilizes Neil1 protein by protecting against proteasomal degradation. We also show that the N-terminal region of mouse Rad9 interacts with Neil1 protein, and this binding is important for Neil1 stability and greater glycosylation activity. Our results thus show for the first time a novel function for RAD9 in regulating NEIL1 and contributing to BER.

## MATERIALS AND METHODS

### Cell lines and culture conditions

Mouse embryonic stem (mES) cells having different status of *Rad9* (*Rad9*^+/+^*, Rad9*^−/−^ and *Rad9*^−/−^ ectopically expressing mouse *Rad9^+^* or human *RAD9*^+^) used for this study were described (30). All the mES cell lines were grown in knockout-DMEM (Invitrogen) supplemented with 15% ES-cell qualified fetal bovine serum (FBS), 0.1 mM non-essential amino acids, 2 mM L-glutamine, 100 μM β-mercaptoethanol, 100 U/ml penicillin/streptomycin and 1 U/ml ESGRO (Leukemia inhibitory factor, LIF, from Millipore). Tissue culture dishes were coated with 0.1% gelatin solution (Millipore) before cell plating. Human prostate cancer cell lines, DU145 and PC-3, were grown in RPMI medium supplemented with 10% FBS and 100 U/ml penicillin/streptomycin. DU145-sh*RAD9* and PC-3-sh*RAD9* cells used for this study were described and grown in a similar manner (31,32). All cell lines were cultured at 37°C, 5% CO_2._

### Plasmid construction and generation of cell lines

Mouse *Neil1* and human *NEIL1* ORF clones were purchased from Origene (#MC211355) and Genecopoeia (#A3833), respectively. Mouse *Rad9* cDNA was generated from mES cells by reverse transcription, using primer pair 5′-GCGCGATCGCCATGAAGTGCCTGATCACC-3′ (forward) and 5′-GCACGCGTCCCTTCACCATCACTGTGTT-3′ (reverse). *Rad9* cDNA was digested by *Asis*1 and *Mlu*1 (restriction enzyme sites underlined in the listed primer pairs) and inserted into pCMV6-AC-DDK-His vector, linearized by the same enzymes. Constructs were transfected into *Rad9*^−/−^ mES and DU145-sh*RAD9* cells using lipofectamine 2000 (Life Technologies), according to the manufacturer's instructions. Selection medium, containing G418 sulphate (100 μg/ml, Cellgro), replaced standard growth medium 48 h post-transfection. Drug-resistant colonies were isolated after two weeks, and the abundance of targeted protein was examined by western analysis using anti-FLAG antibody (Sigma # A8592). Truncated *Rad9* cDNAs were polymerase chain reaction (PCR)-amplified from full-length cDNA using primer pairs 5′-GCGCGATCGCCATGAAGTGCCTGATCACC-3′ (forward) and 5′-GCACGCGTACATGAGTCTTGCTCTAAGA-3′ (reverse) for the N-terminal region (encoding 1–270 aa), and 5′-GCGCGATCGCCATGTGTTCCCAGGGCCCGTGT-3′ (forward) and 5′-GCACGCGTCCCTTCACCATCACTGTGTT-3′ (reverse) for the C-terminal region (encoding 270–389 aa), then inserted into pCMV6-AC-DDK-His as described above. *Rad9*^−/−^ mES cells ectopically expressing truncated *Rad9* cDNAs were generated by transfection. The restriction enzyme digestion map and DNA sequence of all cloned inserts were accessed to confirm correctness, before introduction into cells.

### Clonogenic assay

Five hundred cells were seeded onto each well of a 6-well plate and allowed to attach overnight. For UV irradiation, cells were washed with 1X phosphate buffered saline (PBS), and then irradiated with the indicated doses from a 254 nm UV lamp. After exposure, cells were returned to complete medium. For γ-irradiation, cell culture medium was replaced with fresh medium before exposure at the indicated doses by a Gammacell 40 ^137^Cs irradiator (0.8 Gy/min). For menadione treatment, cells were washed and incubated with indicated concentrations of the drug in serum free medium for 1 h at 37°C, followed by washing with 1X PBS and return to complete medium. In all cases, cells were incubated post-treatment for 7 days, washed once with 1X PBS, fixed with methanol and then stained with 0.5% crystal violet for colony counting. Three independent experiments were performed, with duplicate samples tested in parallel for each dose.

### Glycosylase incision assay using oligonucleotides with modified bases in mES whole cell extracts

Single lesion modifications, including 8-oxo-dG, 5-hydroxy-Uracil, etheno adenosine or an abasic/apurinic site, were incorporated at position 10 (bold font) of the 24-mer oligonucleotide 5′-GAACTAGTG**G**ATCCCCCGGGCTGC-3′ (Midland Certified Reagent Co., Midland, TX, USA). The abasic/apurinic alteration was made using the AP site analogue, furan. All oligonucleotides were 5′ end-labeled with γ-^32^P-ATP (PerkinElmer), and annealed with the complementary strand, as described (33). An oligonucleotide with the same sequence but devoid of modifications was used as control. The incision reaction was carried out in a 25 μl mixture containing 0.2 nMol oligonucleotide duplex, 1 μg of poly (dI-dC) competitor, 20 mM HEPES-KOH, pH 7.8, 100 mM KCL, 5 mM DTT, 5 mM EDTA, 2 mM MgCl_2_ and up to 50 μg of whole cell extract, prepared by routine methods (34). Incision reactions for the abasic substrate were carried out in a similar manner as described above, except no MgCl_2_ was added either during the extract preparation or to the reaction buffer. The mixture was incubated at 37°C for the indicated times, then the reaction was terminated by addition of 1 μl of 10% SDS and 1 μl of 5 mg/ml proteinase K, followed by incubation for 10 min at 55°C. DNA oligonucleotides were precipitated by addition of 4 μl of 5 mg/ml of Glycogen (Ambion), 10 μl of 10 M ammonium acetate and 150 μl of cold ethanol, followed by overnight incubation at −20°C. Samples were centrifuged at 12 000X g for 30 min at 4°C, then washed with 200 μl of 70% ethanol. Pellets were dried and resuspended in 10 μl of formamide loading dye (5% EDTA, 0.025% bromophenol blue, 0.025% xylene cyanol in 95% formamide). Then, samples were fractionated by 18% denaturing Urea-PAGE. Reaction products were visualized by autoradiography and quantified using image-J software.

### Chromatin immunoprecipitation and western blotting

Chromatin immunoprecipitation (ChIP) was performed as described, but with minor modifications (35). Chromatin from DU145 and mES cells was cross-linked by adding 1% formaldehyde in PBS at 37°C for 30 min; then the reaction was stopped by addition of 125 mM glycine. DNA was sheared to 300–1000 bp by sonication. Debris was removed by centrifugation at 16 000X g for 10 min at 4°C, and the supernatant was incubated with anti-Rad9 (Abcam #ab70810) or rabbit IgG (Vector Laboratories Inc. # I-1000) antibodies and protein A/G beads at 4°C overnight. The beads were washed 1X with each of the following buffers: TSE I, 20 mM Tris-HCl (pH 8.1), 150 mM NaCl, 2 mM EDTA, 0.1% SDS, 1% Triton X-100; TSE II, 20 mM Tris-HCl (pH 8.1), 500 mM NaCl, 2 mM EDTA, 0.1% SDS, 1% Triton X-100; ChIP buffer III, 10 mM Tris-HCl (pH 8.1), 0.25 M LiCl, 1 mM EDTA, 1% NP-40, 1% deoxycholate and TE, 10 mM Tris-HCl (pH 8.1), 1 mM EDTA. Chromatin was then eluted and reverse cross-linked by incubation at 65°C. The DNA was purified using the Qiagen PCR purification kit and employed as template for PCR. Six primer pairs spanning the *NEIL1* or *Neil1* promoter were employed. Primer sequences are listed in Supplementary Table S1.

Proteins for western analysis were extracted from DU145, PC-3 and mES cells, with RIPA buffer (50 mM Tris-Cl, pH7.5, 150 mM NaCl, SDS 0.1%, Sodium deoxycholate 0.5%, NP-40 1% plus protease inhibitors). After centrifugation at 14 000X g, protein in supernatants was quantified by the Bradford method (36). Cell lysates (25 μg/lane) were fractionated by sodium dodecyl sulphate-polyacrylamide gel electrophoresis (SDS-PAGE). Proteins were transferred to polyvinylidene fluoride membranes and blocked with 5% nonfat dry milk in 1X TBST (0.9% NaCl, 0.1% Tween 20, 100 mM Tris-HCl, pH 7.5). Membranes were then incubated with primary antibodies, anti-Rad9 (BD Biosciences # 611324), anti-Neil1 (Abcam # ab21337) or anti-β-Actin (Sigma-Aldrich # A5441), overnight at 4°C. After three washes with 1X TBST, membranes were incubated with appropriate secondary antibodies for 1 h. After three washes with 1X TBST, peroxidase activity was visualized using Western Lightning Plus-ECL (PerkinElmer) as per the manufacturer's instructions. Gel bands were quantified using Image-J software.

### Immunoprecipitation

Anti-FLAG M2-gel (Sigma-Aldrich # A2220) was used for immunoprecipitation (IP) with mES cells ectopically expressing Rad9 proteins, as well as controls, according to the manufacturer's instructions. For IP of Neil1, 500 μg of pre-cleared mES cell lysates were incubated with anti-Neil1 (Abcam # ab21337) or rabbit IgG (Vector Laboratories Inc. # I-1000) antibodies overnight, followed by addition of Protein A/G agarose beads to the mixture. Unbound proteins were separated by centrifugation at 5000X g for 2 min at 4°C, followed by three washes using binding buffer (25 mM Tris-HCl, pH7.4, 150 mM NaCl, 1 mM EDTA, 1% NP40, 0.5 mM DTT and 5% glycerol). Immunoprecipitated proteins were eluted by adding 25 μl of 2X sample buffer (125 mM Tris HCl, pH6.8, 4% SDS, 20% glycerol and 0.004% bromophenol blue) to each reaction. Controls were treated similarly.

### Generation of luciferase reporter constructs and assaying luciferase activity

Promoter regions of human *NEIL1* (−996 to +81) and mouse *Neil1* (−960 to +81) were produced by PCR amplification from DU145 and mES genomic DNA using, respectively, primer pairs 5′-GCCTCGAGCGCCTGTAATCCCAACACTTTGG-3′ (forward) and 5′-GCAAGCTTGGCGGAAGGAACCGCCAGTACA-3′ (reverse) for human *NEIL1*, and 5′-GCCTCGAGCCCGGGAAAGACAGAGAAACCA-3′ (forward) and 5′-GCAAGCTTCACACACCCACCAAATACCAGC-3′ (reverse) for mouse *Neil1*. Underlined nucleotides in forward primers correspond to *Xho*I restriction sites, and in reverse primers the sequences are *Hind*III sites. Amplified fragments were then cloned into pGL3-Basic vector at *Xho*I and *Hind*III restriction sites, placing the inserts 5' to the luciferase reporter gene within the plasmid. Deletion constructs lacking the p53-binding site in each promoter were made by PCR amplification from the aforementioned plasmids, and then ligated into insertless pGL3-Basic as above. Mutations in the p53-binding sites in the human *NEIL1* promoter (−996 to +81) were produced by the QuikChange Site-Directed Mutagenesis Kit, according to the manufacturer's protocol (Stratagene # 200519). Primers used for mutagenesis are as follows: 5′-GAAATCTGGATGTTTAGATGATATTAAG-3′ and 5′-CTTAATATCATCTAAACATCCAGATTTC-3′ for the first p53-binding site (−702 to −693), and 5′-GTTACTGTTGGCTTTTTCGTGTGGCTCACTTC-3′ and 5′-GAAGTGAGCCACACGAAAAAGCCAACAGTAAC-3′ for the second one (−663 to −654), respectively. The primers were designed so that the first p53-binding site (−702 to −693) will be deleted, and point mutations will be created at the second p53-binding site (−663 to −654; 5′-GGGCATGGTG-3′ to 5′-TTTTCGTGTG-3′). Promoter constructs were confirmed for correctness by DNA sequence analysis.

### RNA isolation and qRT-PCR

Total RNA from mES, DU145 and PC-3 cells was isolated using the miRCURY RNA Isolation Kit (EXIQON), according to the manufacturer's instructions. Total RNA (1 μg) was reverse transcribed using the SuperScript III first strand synthesis system (Life Technologies), with oligo (dT) primer. Equal amounts of cDNA were subjected to real-time PCR using SYBR-Green PCR master mix (Applied Biosystems) in an ABI 7300 real-time PCR system. The following primer pairs were employed: *NEIL1*, 5′-GCCCTATGTTTCGTGGACATC-3′ (forward) and 5′-CGCTAGGTTTCGTAGCACATTC-3′ (reverse); *Neil1*, 5′-GCTTGCCCTTTGCTTCGTAGA-3′ (forward) and 5′-CCCGCAGATAGTTGCCAATG-3′ (reverse); *RAD9*, 5′-TCTGCCTATGCCTGCTTTCTCT-3′ (forward) and 5′-AGCGGAAGACAGACAGGAAAGAC-3′ (reverse); *Rad9*, 5′-GGCTGTCCATTCGCTATCCC-3′ (forward) and 5′-GTGGGGCAAAAAGGAGCAG-3′ (reverse); and *β-Actin*, 5′-GAGCTAGGAGCTGCCTGAC-3′ (forward) and 5′-AGCACTGTGTTGGCGTACAG-3′ (reverse).

## RESULTS

### Rad9 depletion reduces Neil1 protein abundance in mouse ES cells and similarly in human prostate cancer cells

**Figure 1.**
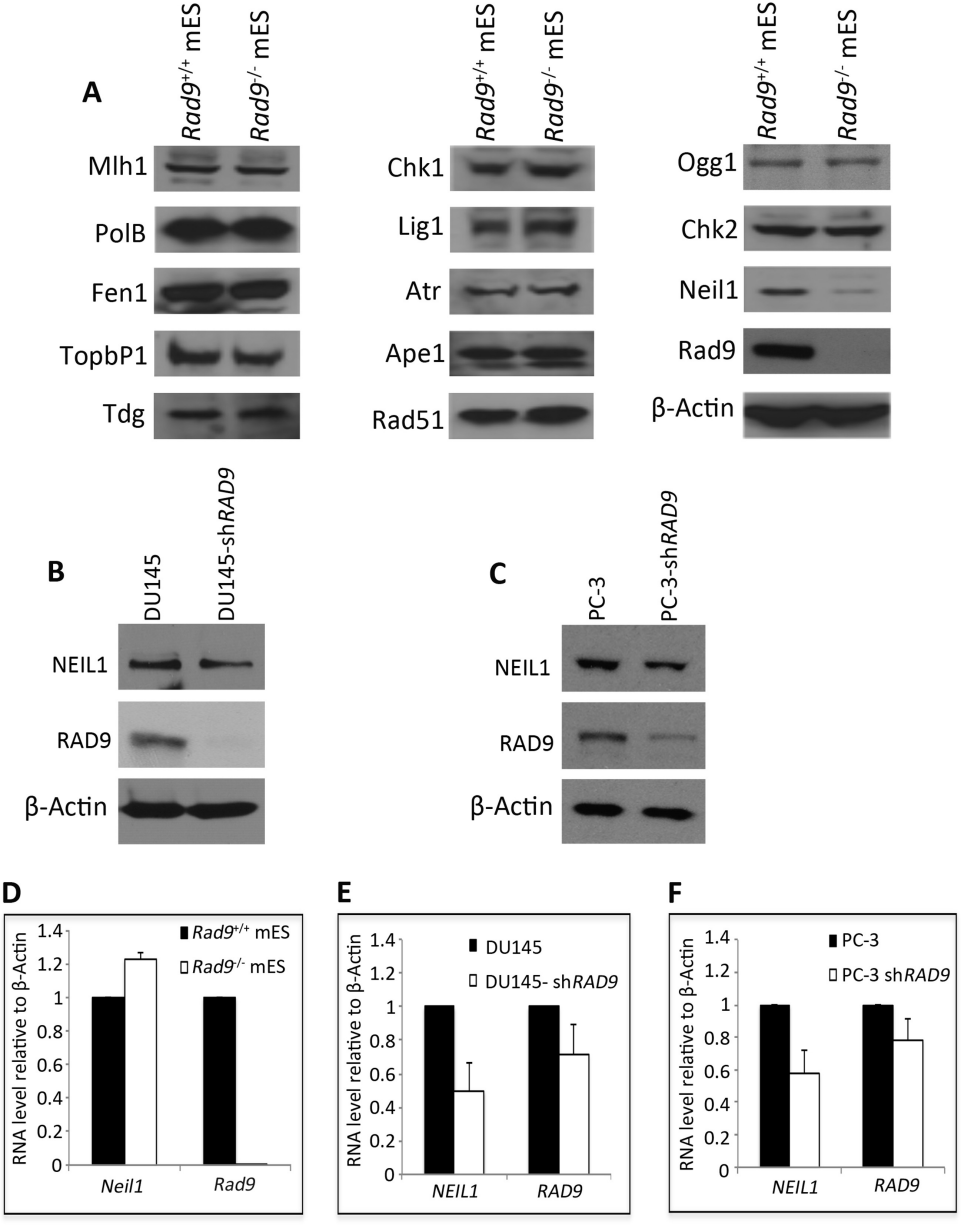
Neil1 protein and RNA abundance in mES and human prostate cancer cells with inherent or reduced levels of Rad9 protein. (**A**) Immunoblotting analyses used to measure indicated DDR proteins in *Rad9^+/+^* and *Rad9^−/−^* mES cells. β-Actin, loading control. (**B**) Immunoblotting used to measure NEIL1 protein abundance in DU145 cells with inherent or sh*RAD9* knocked down RAD9 levels. β-Actin, loading control. (**C**) Same as B, but PC-3 cells were examined. (**D**) qRT-PCR used to assess *Neil1* and *Rad9* RNA levels in *Rad9*^+/+^ and *Rad9*^−/−^ mES cells, plotted relative to β-Actin levels. (**E, F**) Same as D, but using DU145 and PC-3 cells, respectively, with inherent or shRNA-reduced levels of RAD9. Error bars in D, E and F represent standard deviation of three independent experiments.

Rad9-deficient mES cells are very sensitive to a wide variety of DNA damaging agents relative to wild-type controls (30). It is also known that human RAD9 can regulate levels of other proteins by controlling transcription of corresponding, specific downstream target genes, such as *p21* (33), or by influencing protein stability, as for DDB2 and ITGB1 (21,32). We examined levels of several DDR proteins in mES cells either proficient or null for Rad9, to access whether it might impart DNA damage resistance due to regulation of the abundance of other proteins. Out of 13 DDR proteins tested, only Neil1 level was reduced by 4-fold in *Rad9* null cells, compared to the *Rad9^+/+^* control (Figure 1A). Furthermore, we tested NEIL1 abundance in human prostate cancer DU145 and PC-3 cells with inherent or shRNA-reduced RAD9 levels. Relative to parental cell populations and internal levels of β-Actin, shRNA reduced RAD9 protein abundance by 89% in DU145 and 78% in PC-3 cells (Figure 1B and C). We also observed that when RAD9 level was lowered, NEIL1 protein quantity was reduced by 51% in DU145 and by 43% in the PC-3 populations.

### RAD9 depletion reduces *NEIL1* RNA abundance in human prostate cancer cells but not similarly in mouse ES cells

To test if Rad9 regulates *Neil1* at the RNA level, we measured *Neil1* mRNA abundance in mouse *Rad9^+/+^* and *Rad9^−/−^* ES cells. As indicated in Figure 1D, no *Rad9* RNA was detected in *Rad9^−/−^* cells, as expected since the mutant is a null (30). Furthermore, *Neil1* RNA abundance was not reduced in *Rad9^−/−^*, relative to the *Rad9^+/+^* control. We next performed a similar experiment with human prostate cancer cells. We found that down regulation of *NEIL1* mRNA in DU145-sh*RAD9* and PC-3-sh*RAD9* cells was observed relative to the controls, and that this down regulation was approximately at the same fold change compared to the immunoblot results for NEIL1 protein (Figure 1B, C, E, F). There was only a 30% decrease in *RAD9* mRNA observed in DU145-sh*RAD9* and a 20% decrease in PC-3-sh*RAD9* cells relative to the parental controls. This is expected, as most of the regulation should be at the translational level rather than by transcription repression. These results suggest that Rad9 is involved in post-transcriptional regulation of *Neil1* in mES cells, but in contrast at the transcriptional level in human DU145 and PC-3 cells.

**Figure 2.**
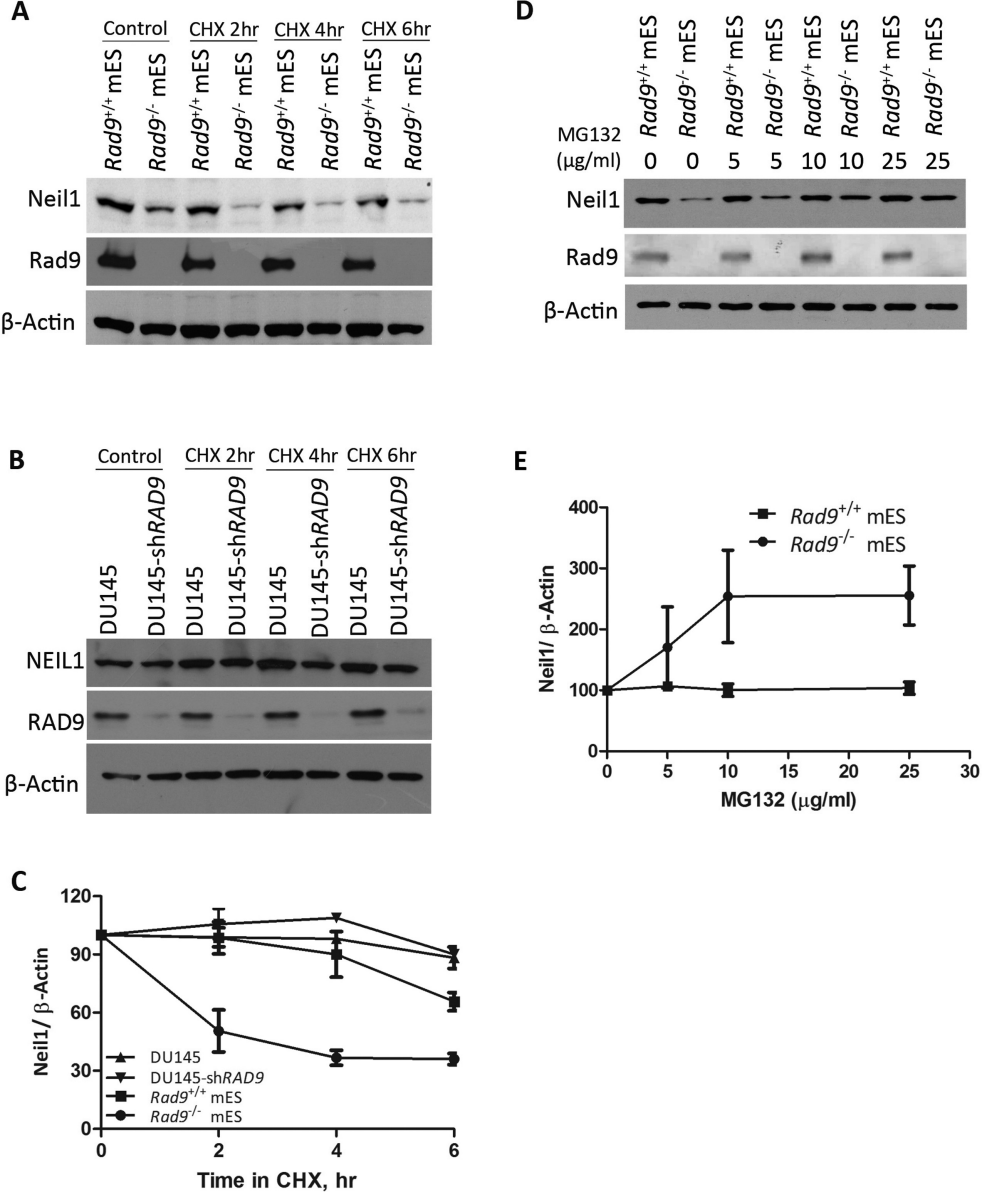
Rad9 controls Neil1 protein stability in mES but not in DU145 cells. (**A**) Neil1 and Rad9 protein levels were detected by immunoblotting in *Rad9^+/+^* and *Rad9^−/−^* mES cells after treating with CHX (50 μg/ml) for indicated time intervals. β-Actin was the loading control. (**B**) Same as A, but using DU145 cells with or without sh*RAD9*. (**C**) Average Neil1 protein level relative to β-Actin was calculated by densitometric measurements of bands from three independent experiments (as in A, B). Error bars represent standard deviation. (**D**) Neil1 and Rad9 abundance was assessed by immunoblotting analyses using *Rad9^+/+^* and *Rad9^−/−^* mES cells grown in the presence or absence of proteasomal inhibitor MG132 at concentrations indicated. β-Actin is the loading control. (**E**) Average Neil1 protein level relative to β-Actin was calculated by densitometric measurements of bands from three independent experiments (as in D).

We reported that RAD9 has a role in mediating IGTB1 protein stability in DU145 cells (32). Therefore, we hypothesized that Rad9 might have a similar role in regulating Neil1 protein stability in mES cells. To test this, we treated mES and DU145 cells with the protein biosynthesis inhibitor cycloheximide (CHX), and then performed western analysis. Neil1 protein level was much lower in *Rad9*^−/-^ mES cells as compared to *Rad9*^+/+^ cells 2 h post-treatment, whereas no change in NEIL1 protein level was observed in DU145-sh*RAD9* cells under the same conditions as compared to the parental control (Figure 2A and B). In *Rad9*^+/+^ mES cells, Neil1 has a half-life of 10 h (Supplementary Figure S1), whereas average densitometry measurements of the bands from three independent experiments confirmed that Neil1 has a shorter half-life (2.5 h) in *Rad9^−/−^* mES cells after CHX application (Figure 2C).

Our results indicate that NEIL1 protein levels are regulated differently in human prostate cancer cells versus mouse ES cells. Proteasomes act as a special regulatory unit in ES cells to prevent incorrect transcription initiation and regularly degrade proteins needed for differentiation (37). To test whether Neil1 is degraded by proteasomes in mES cells in the absence of Rad9, we treated *Rad9*^+/+^ and *Rad9*^−/−^ mES cells with proteasomal inhibitor MG132 at various concentrations for 4 h. An increase in Neil1 protein level was observed in *Rad9^−/−^* mES cells after adding MG132 for 4 h whereas no change in Neil1 level was seen in *Rad9*^+/+^ cells (Figure 2D). Average densitometry measurements of the bands from three independent experiments confirmed that the increase in Neil1 protein level by MG132 is concentration dependent up to 10 μg/ml (Figure 2E). Hence, the shorter half-life of Neil1 protein in the absence of Rad9 in mES cells is due to proteasomal degradation.

### RAD9 binds to the human *NEIL1* promoter, and similar interactions occur in mouse cells

**Figure 3.**
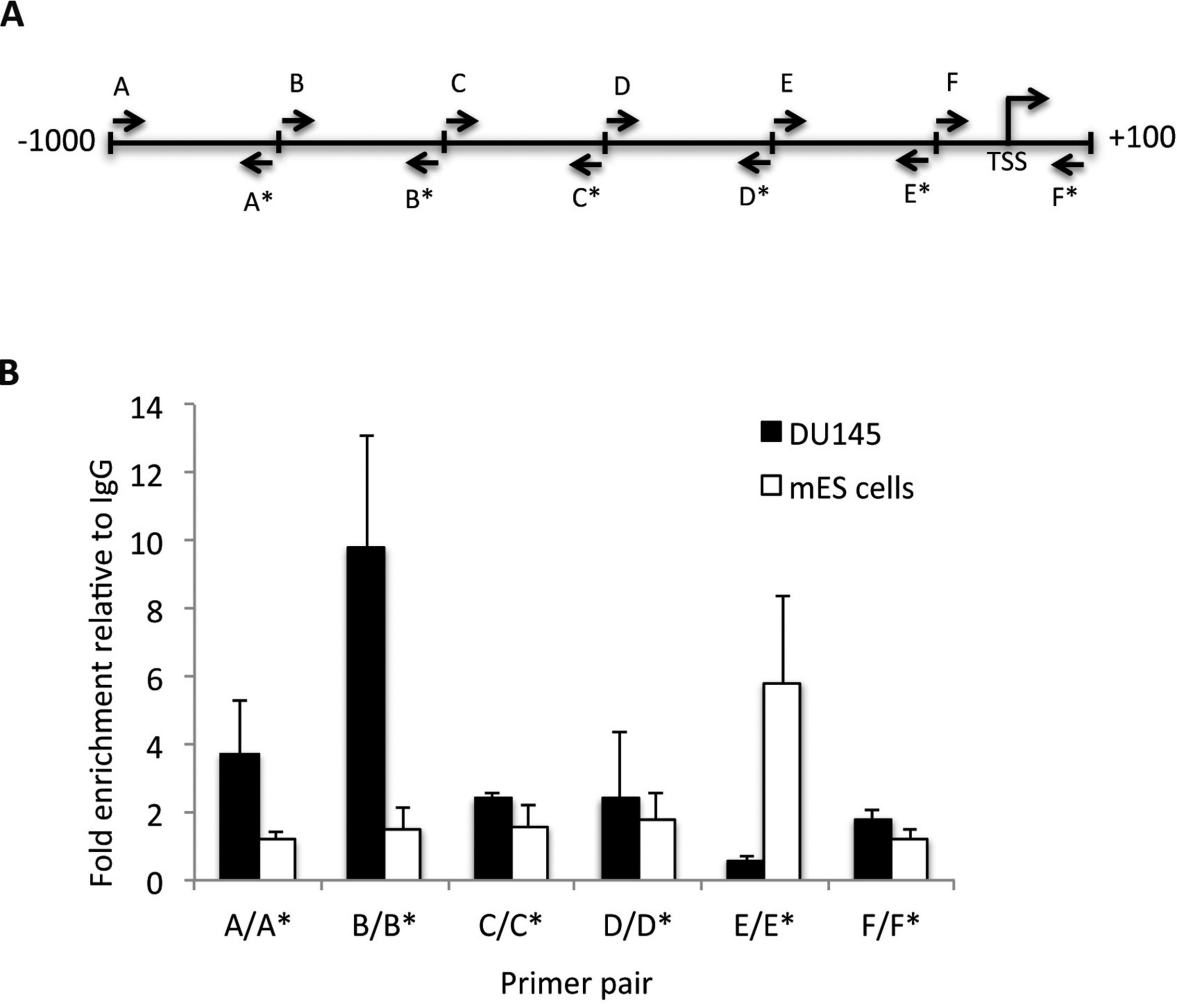
RAD9 protein binds the *NEIL1* promoter. Binding of RAD9 and Rad9 to their corresponding *NEIL1/Neil1* promoter was tested by ChIP-qPCR, using DU145 and mES cells, respectively. (**A**) Schematic representation of *NEIL1/Neil1* promoters with the approximate position of primer pairs used for ChIP-qPCR experiments (see Supplementary Table S1 for primer details); each letter represents the primer pair; asterisk indicates reverse orientation primer of pair. TSS is the transcription start site. (**B**) Fold enrichment of RAD9 or Rad9 relative to IgG in ChIP-qPCR experiments. Error bars represent the standard deviation of three independent experiments.

RAD9 can regulate transcription of *p21* by binding at p53 consensus sequences in its promoter (33). We observed reduced *NEIL1* RNA levels in human prostate cancer cells after RAD9 depletion, and thus wanted to address whether a similar regulatory mechanism was responsible. To test this, ChIP-qPCR analysis was performed to examine if RAD9 can bind the *NEIL1* promoter in DU145 cells, and a similar experiment was conducted with mES cells. It was observed that RAD9 and Rad9 bind *NEIL1* and *Neil1* promoters, respectively, but at different positions relative to corresponding transcriptional start sites (TSS) (Figure 3). For human *NEIL1*, RAD9 binds to the upstream promoter region (−821 to −641 bp), whereas in mouse binding occurs between −281 and −100, very close to the transcription start site. By pair-wise alignment of both bound sequences and TRANSFAC analysis, we did not find any common motif or putative transcription factor-binding site, except for p53 consensus-like motifs (standard: 5′-PuPuPuC(A/T)(T/A)GPyPyPy-3′) (38). In the mouse *Neil1* promoter, there are two sites with an 80% match (−225 to −216, 5′-GAGCAAGACA-3′; −68 to −59, 5′-GAACAAGACA-3′), but the second one is outside of the ChIP positive promoter region (−281 to −100). The human promoter has two p53 sites within the ChIP positive region. The first is a 90% match (−702 to −693, 5′-GGACTAGCTA-3′) and the second is 80% identical (−663 to −654, 5′-GGGCATGGTG-3′). Therefore, as per *p21* regulation (33), it is possible that RAD9 is controlling at least human *NEIL1* expression by binding to a p53 consensus site in DU145 cells. As we did not see any difference in *Neil1* mRNA levels in mES cells sufficient or null for *Rad9*, such regulation is not expected in these cells. Therefore, binding of RAD9 to the *NEIL1* promoter is not cell specific, which is in contrast to RAD9-dependent transcription regulation.

**Figure 4.**
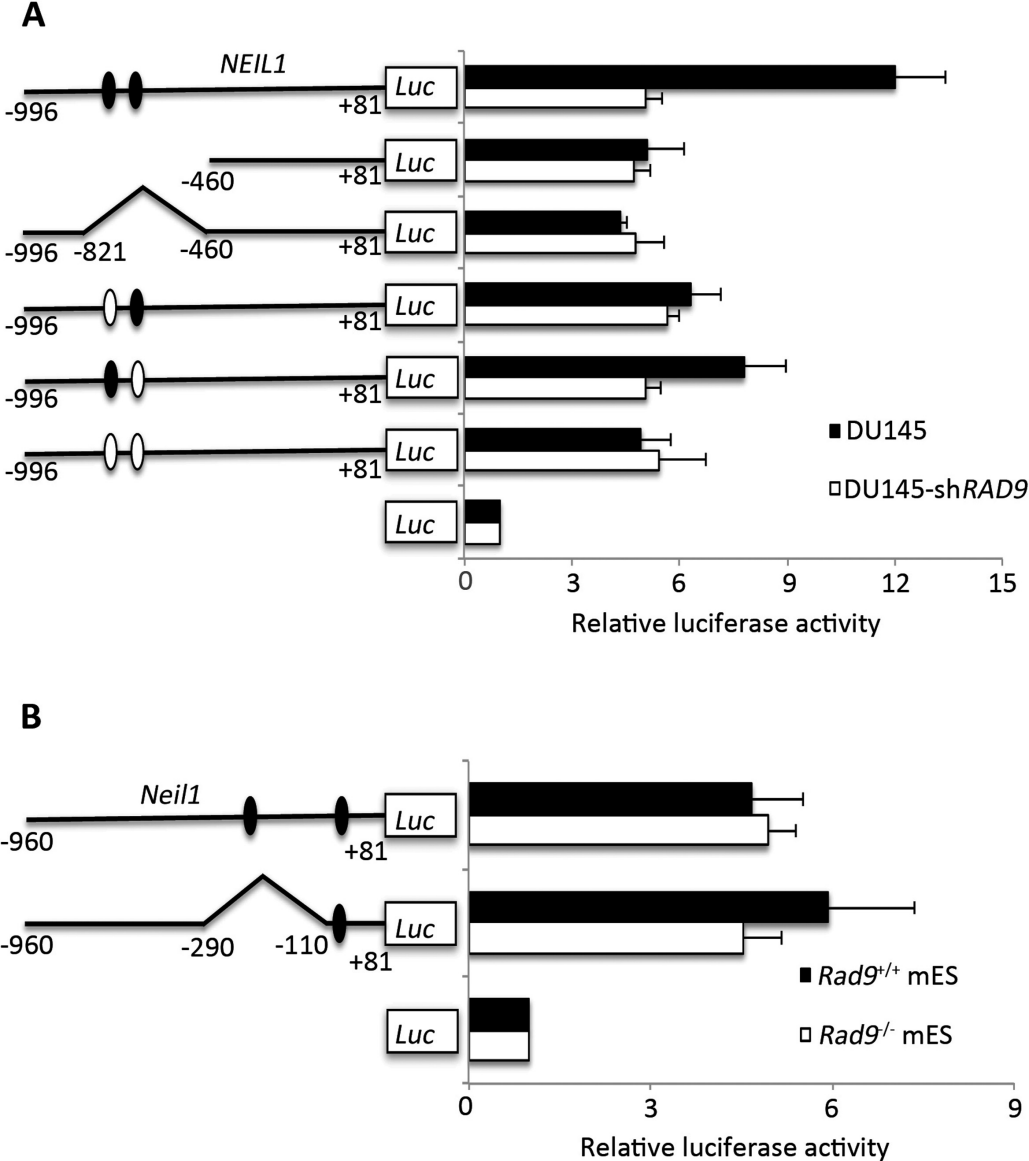
*NEIL1* promoter-luciferase reporter activity in DU145 and mES cells with inherent or reduced levels of RAD9. Chimeric constructs of the *NEIL1* promoter-luciferase reporter are schematically represented on the Y-axis. The X-axis indicates luciferase activity as fold above values obtained for the promoterless vector, pGL-Basic. (**A**) Human *NEIL1* promoter sequence. DU145 (dark bar), DU145-sh*RAD9* (light bar) host cells. (**B**) Mouse *Neil1* promoter sequences. mES *Rad9^+/+^*(dark bar), *Rad9^−/−^* (light bar) host cells. Error bars represent the standard deviation of three independent experiments. Luc, luciferase. Numbers on constructs in Y-axis correspond to nucleotide positions in promoters relative to the start of transcription. Dark ovals represent intact p53-binding sites; light ovals indicate mutation sites. Pointed regions of promoters contain RAD9 binding sequences, as per the Chip-qPCR data, and are deleted.

To extend these observations, we cloned mouse *Neil1* and human *NEIL1* promoter regions, with or without p53-binding sites, upstream of the luciferase reporter gene, and transfected these constructs into mES and DU145 cells either fully expressing or lacking Rad9 and RAD9, respectively. Mouse ES cells do not express active p53 (39) and DU145 cells have two point mutations in the *p53* gene, which cause formation of a temperature sensitive (TS) mutant of the protein (40). Later observations confirmed that this TS form of p53 could not transactivate its usual downstream target, *p21*, in parental DU145 cells (41). Therefore, p53-mediated activation of the luciferase reporter in DU145 cells is not a consideration. As expected, we observed a 50% decrease in reporter activity in DU145 cells either lacking RAD9 or the p53-binding site, relative to controls (Figure 4A). However, the same was not observed in mES cells (Figure 4B), as those either having or lacking Rad9 showed equivalent luciferase activity. In addition, deleting the p53 consensus sequence in the reporter construct did not alter luciferase activity. To confirm these observations and also test whether cell type or the origin of the promoter is important for function, we transfected human *NEIL1* promoter constructs into mouse cells (mES) and mouse *Neil1* promoter constructs into human cells (DU145). We found that RAD9-dependent *Neil1* regulation was dependent on the p53 consensus sequences only in DU145 cells, and human *NEIL1* promoter activity was not dependent on Rad9 or p53 consensus sequences when assayed in the mES cells (Supplementary Figure S2). This suggests that, although RAD9 binds to both mouse and human promoters, mES cells lack additional factors essential for *Neil1* or *NEIL1* transcription regulation.

### DNA damaging agent sensitivity of DU145 and mES cells with different RAD9/Rad9 and NEIL1/Neil1 levels

**Figure 5.**
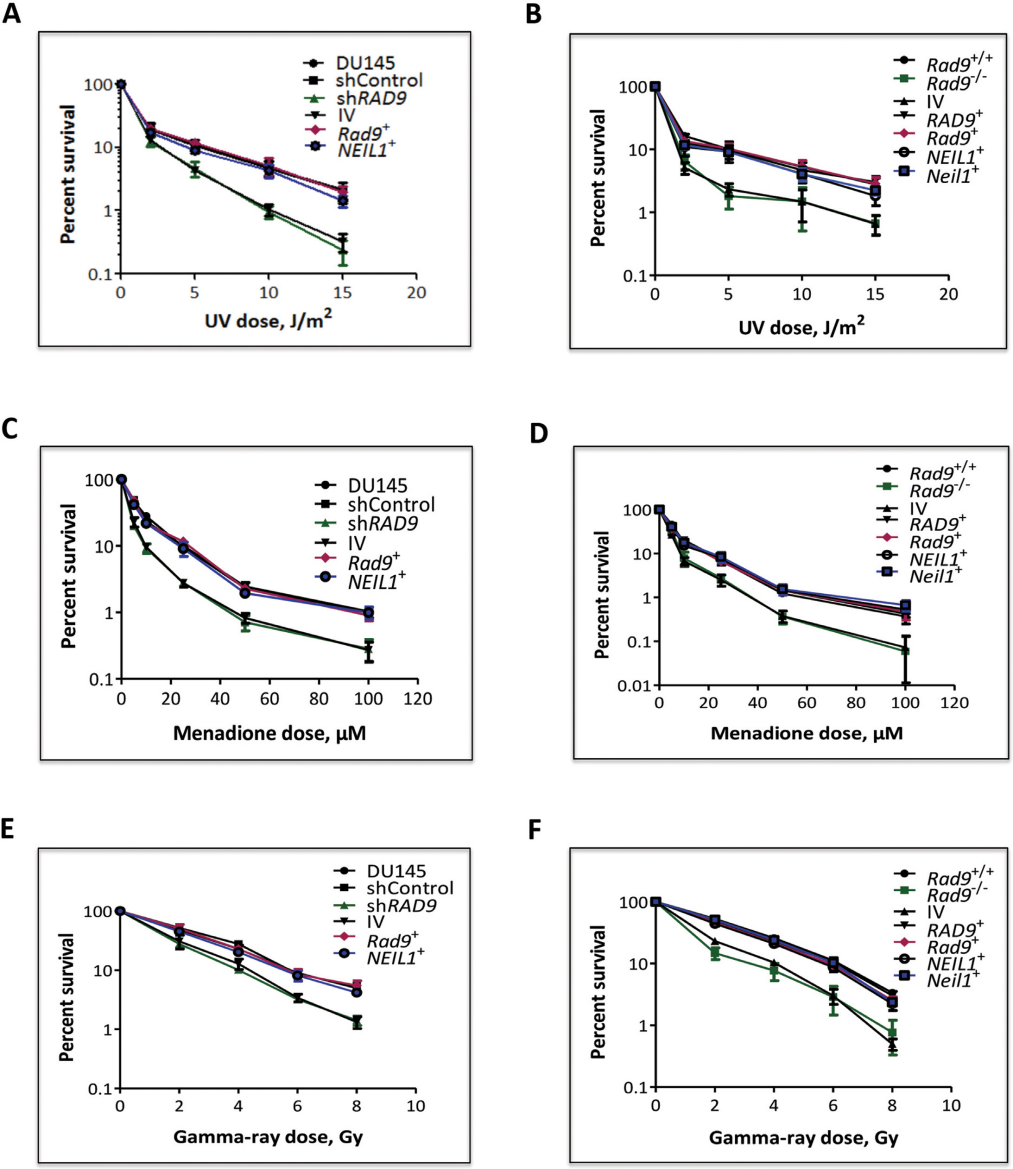
Clonogenic survival of mES and DU145 cells with varying status of *Rad9* after UV, menadione, and gamma-ray treatment. (**A, B**) Sensitivity of cells to 254 nm UV light. (**C, D**) Sensitivity of cells to menadione. (**E, F**) Sensitivity of cells to gamma rays. A, C and E, parental DU145 cells or those with shControl or sh*RAD9*, and the latter with insertless pCMV6-AC-DDK-His vector, or ectopically expressing *Rad9^+^* or *NEIL1^+^*. B, D and F, mES cells *Rad9^+/+^, Rad9^−/−^*, or the latter with insertless pCMV6-AC-DDK-His vector, or ectopically expressing *RAD9^+^*, *Rad9^+^*, *NEIL1^+^* or *Neil1^+^*. Percent survival after each treatment was calculated as the number of colonies formed in treated versus mock-treated populations, times 100. Points are the average of three independent trials, each with two dishes per point. Error bars, standard deviation.

We showed that mES cells lacking Rad9 are more sensitive to UV, gamma rays and hydroxyurea than controls (30). As indicated in Figure 1, we demonstrated that Rad9 depletion causes a reduction in levels of Neil1, which functions in BER (42) and nucleotide excision repair (43). Therefore, we tested whether Neil1 can be a downstream target of Rad9 responsible for DNA damage resistance. We ectopically expressed *NEIL1/Neil1* or *RAD9/Rad9* in *Rad9*^−/−^ mES cells, and *Rad9* as well as *NEIL1* in DU145 cells containing sh*RAD9*. Ectopic expression of *Rad9* or *RAD9* in mouse *Rad9^−/−^* ES cells increased Neil1 protein level (Supplementary Figure S3A). Similarly, *Rad9* expression in DU145-sh*RAD9* cells increased NEIL1 protein abundance (Supplementary Figure S3B). Colony forming ability of mES and DU145 cells with different levels of Rad9/RAD9 and Neil1/NEIL1 exposed to UV, gamma rays, or menadione was assessed (Figure 5). Cells either lacking or having reduced Rad9/RAD9 abundance were more sensitive to all three agents, relative to controls. As expected, ectopic expression of *Rad9* was able to complement the sensitivity of DU145-sh*RAD9* cells, and *Rad9* as well as *RAD9* expression enhanced resistance to *Rad9^−/−^*mES cells. Ectopic expression of *NEIL1* in the DU145-sh*RAD9* cells restored near parental levels of resistance (Figure 5A, C, E), which was also observed when *Neil1* or *NEIL1* was expressed in the *Rad9^−/−^* mES cells (Figure 5B, D, F). The latter indicates that NEIL1 is downstream of RAD9 and, predictably, increasing just NEIL1 level in RAD9-deficient cells can restore cellular resistance to DNA damage.

### Glycosylase activity (incision) on oligonucleotides containing single base modifications in mES and DU145 whole cell extracts

**Figure 6.**
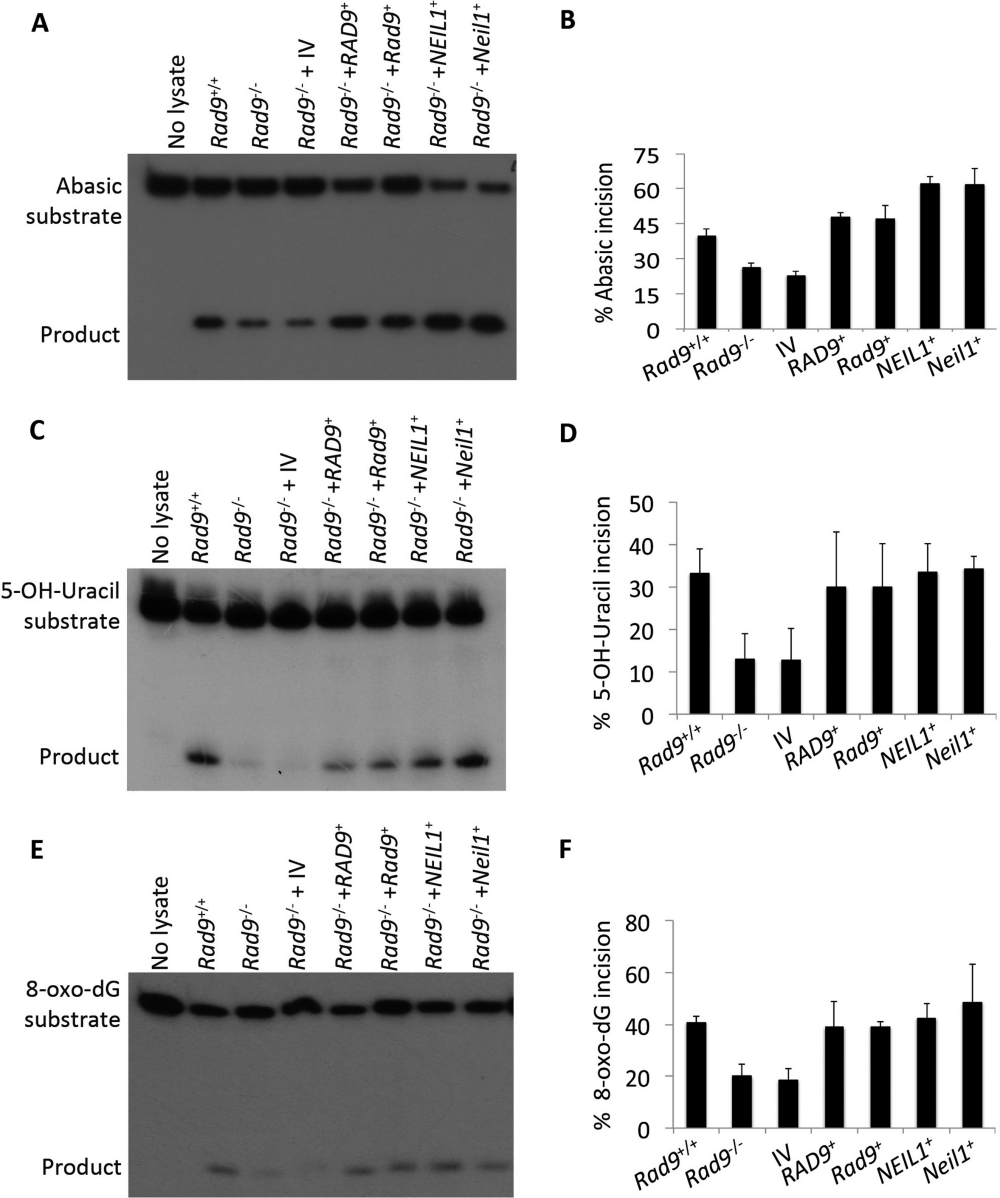
Glycosylase activity on different substrates in extracts from mES cells with varying Rad9 and Neil1 status. Glycosylase activity (incision) was measured by an *in vitro* assay using a 24-mer oligo substrate containing either abasic (**A, B**) 5-OH-Uracil (**C, D**) or 8-oxo-dG (**E, F**) modifications, coupled with extracts from mES cells, either *Rad9^+/+^, Rad9^−/−^*, or the latter with insertless pCMV6-AC-DDK-His vector (IV), or ectopically expressing *RAD9^+^*, *Rad9^+^*, *NEIL1^+^* or *Neil1^+^*. Panels A, C, E: *in vitro* incision assay showing 24-mer oligo substrate and 10-mer product. Average percent incision from three independent experiments shown in panels B, D, F; error bars, standard deviation.

We demonstrated that RAD9 is responsible for NEIL1 regulation, and cells with reduced levels of either protein are more sensitive to several DNA damaging agents, relative to controls. We next tested whether, as predicted, glycosylase activity is reduced in RAD9-deficient cells. We performed an *in vitro* incision assay using extracts from various cell lines and a double-stranded 24-mer oligonucleotide substrate, either intact or with any of four modifications (abasic/apurinic, 5-OH-Uracil, 8-oxo-dG and etheno adenosine) at position 10. As indicated in Figure 6, incision activity on the substrates with abasic (Figure 6A and B), 5-OH-Uracil (Figure 6C and D) or 8-oxo-dG (Figure 6E and F) is lower in *Rad9*^−/−^ mES cells, compared to the *Rad9*^+/+^ control. No difference was observed in the incision activity measured using *Rad9*^−/−^ and *Rad9*^+/+^ whole cell extracts on the oligonucleotide containing etheno adenosine (Supplementary Figure S4A), which is known to be repaired by N-glycosylases and not a substrate for NEIL1 glycosylase (11,44). In addition, no incision activity was observed on control, unmodified oligonucleotides using lysates from all the cell lines (Supplementary Figure S4B), which confirms assay specificity. Base excision and AP lyase activity are tightly coupled for NEIL members (12). To ensure the lyase activity is specific to Neil1 rather than due to Ape1 endonuclease activity, although Ape1 protein level is equivalent in Rad9^+/+^ and Rad9^−/−^ mES cells (Figure 1A), reactions with abasic substrate were performed in magnesium-free conditions as described in Materials and Methods. Mg^2+^ is required for APE1 function (45) whereas NEIL1 activity is independent of Mg^2+^ levels (46). A time course curve for abasic incision using wild-type mES whole cell lysates either in the presence or absence of MgCl_2_ showed that MgCl_2_ increases the amount of incision product at least by 2-fold (Supplementary Figure S5). These results suggest that the incision product observed when MgCl_2_ is present might result from the sum of Neil1 and Ape1 activities. In the absence of MgCl_2_, incision is due solely to Neil1. Ectopically expressing *RAD9*/*Rad9* or *NEIL1*/*Neil1* restored incision activity to the *Rad9*^−/−^ mES cell extracts. Pooling the data from three independent trials, we observed that mES cells lacking Rad9 showed a 37% decrease in abasic site incision, a 60% decrease in 5-OH-Uracil site incision and a 50% decrease in 8-oxo-dG site incision (Figure 6B, D, F, respectively). A similar pattern of incision activity was also demonstrated in DU145 cell extracts with different RAD9/Rad9 or NEIL1 protein levels (Supplementary Figure S6). DU145 cells expressing shRNA against *RAD9* showed a 59% decrease in incision at the abasic site, a 60% decrease at 5-OH-Uracil and a 43% decrease at 8-oxo-dG, relative to controls (Supplementary Figure S6B, D, F, respectively). These results indicate that the decreased amounts of Rad9/RAD9 cause a reduction in Neil1/NEIL1 protein levels and that leads to defective BER, as well as enhanced sensitivity to certain DNA damaging agents.

### N terminal region of Rad9 interacts with Neil1

**Figure 7.**
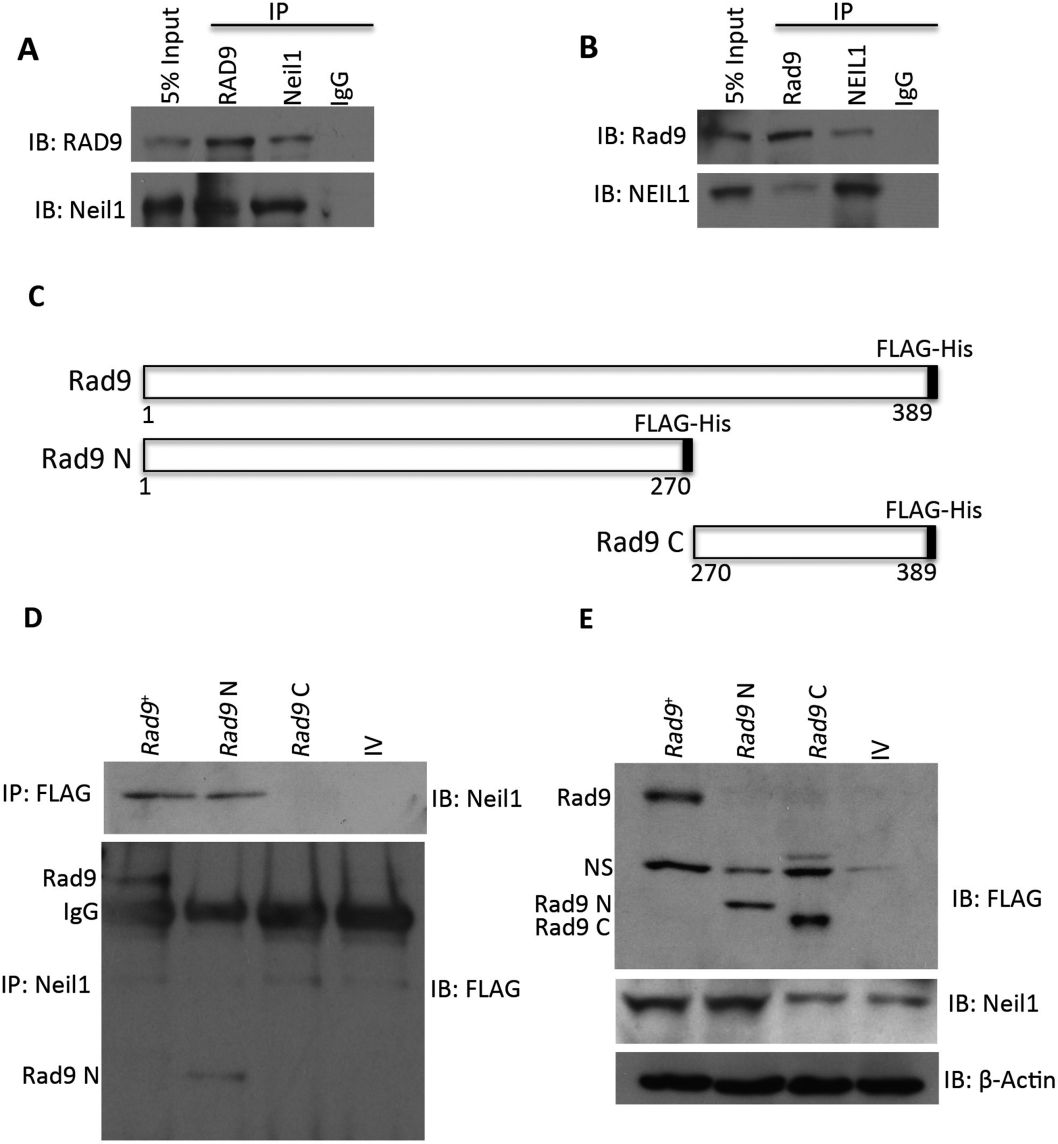
Deletion analysis used to determine the region of mouse Rad9 protein involved in Rad9–Neil1 binding. (**A**) Ectopically expressed RAD9 and endogenous Neil1 were immunoprecipitated from *Rad9*^−/−^ mES cells individually and tested for binding to the other. (**B**) Same as A, but in DU145-sh*RAD9* cells ectopically expressing FLAG-Rad9. (**C**) Graphic depiction of amino acids encoded by inherent or truncated *Rad9* cloned into pCMV6-AC-DDK-His vector, which adds a C-terminal FLAG-His tag to each protein; numbers represent the amino acid positions. Dark box at C-terminal end represents FLAG-His tag. Rad9, full length; Rad9 N, amino-end fragment; Rad9 C, carboxy-end fragment. (**D**) Binding of intact or deletion mutants of Rad9 to Neil1, shown by IP either with anti-FLAG (upper panel) or anti-Neil1 (lower panel) antibody. (**E**) Immunoblot showing abundance of Rad9 and Neil1 proteins in whole cell extracts from IP experiments in panel B. β-Actin was used as loading control. Rad9 N, amino-terminal fragment; Rad9 C, carboxy-terminal fragment; NS, non-specific; IP, immunoprecipitation; IB, immunoblot.

**Figure 8.**
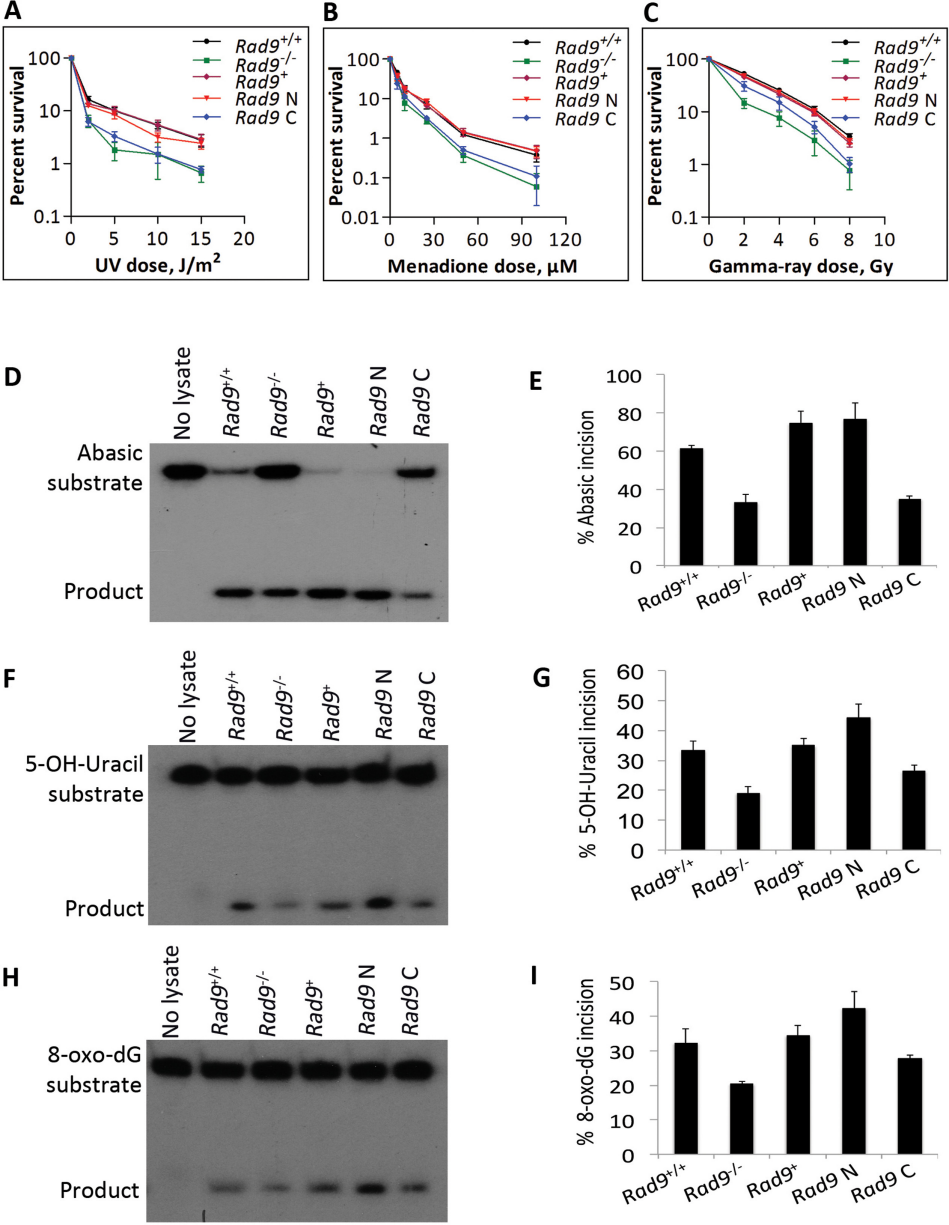
The N-terminal region of Rad9 is important for conferring DNA damage resistance and efficient glycosylase activity to mES cells. Cell survival after exposure to (**A**) 254 nm UV light, (**B**) menadione and (**C**) gamma rays. Cells: mES cells bearing *Rad9^+/+^*, *Rad9^−/−^*, and the latter ectopically expressing *Rad9^+^*, *Rad9* N (encoding N-terminal region of Rad9 from aa 1 to 270) or *Rad9* C (encoding C-terminal region of Rad9 from aa 270 to 389). Points are the average of three independent trials, each with duplicate dishes; error bars represent standard deviation. Glycosylase activity (incision) was measured by an *in vitro* assay using a 24-mer oligo substrate containing either abasic (**D, E**), 5-OH-Uracil (**F, G**) or 8-oxo-dG (**H, I**) modifications in extracts from *Rad9*^+/+^or *Rad9*^−/−^ mES cells or the later containing *Rad9*^+^, *Rad9* N or *Rad9* C. Panels D, F, H: denatured polyacrylamide gel showing *in vitro* incision of the 24-mer substrate and the 10-mer product. The average percent incision of substrate from three independent experiments is shown in panels E, G and I. Error bars, standard deviation.

NEIL1 physically interacts with the RAD9-HUS1-RAD1 complex, as well as with each component individually (29). We showed using clonogenic survival and BER assays that deficiencies in human NEIL1 and RAD9 can be complemented by their respective mouse homologues. This is predictable as the corresponding proteins are >80% identical. The cross species interactions of NEIL1-Rad9 and Neil1-RAD9 were further demonstrated by co-IP (Figure 7A and B). A stronger interaction was observed in mES cells as compared to DU145 cells, suggesting that the mouse cells might contain additional factors needed for enhancing this interaction. It was reported that the C-terminal region of NEIL1 is involved in RAD9–NEIL1 binding (29), and another study revealed that it is involved in intra-molecular interactions that promote NEIL1 stability (47). Our data suggest that the Rad9–Neil1 interaction protects Neil1 from proteasomal degradation in mES cells. Therefore, we sought to identify the region of Rad9 involved in this interaction. We ectopically expressed full-length mouse *Rad9* (*Rad9*^+^), as well as the N-terminal region encoding 1–270 aa (*Rad9* N), and the C-terminal region encoding 270–389 aa (*Rad9* C) in *Rad9*^−/−^ mES cells such that each had a FLAG and HIS tag at its C-terminal end (Figure 7C). By IP, either with anti-FLAG M2 beads or anti-Neil1 antibody, we found that both Rad9^+^ and Rad9 N, but not Rad9 C, are engaged in the Rad9–Neil1 interaction (Figure 7D). Next, we asked whether this interaction has any effect on Neil1 stability and sensitivity of cells to DNA damaging agents. A 60% decrease in Neil1 protein level was observed in cells bearing *Rad9* C, compared to those having *Rad9*^+^ or *Rad9* N (Figure 7E). As assessed by colony formation, cells producing *Rad9* N, relative to those containing *Rad9* C, are more resistant to UV (Figure 8A), menadione (Figure 8B) and gamma rays (Figure 8C), and nearly equal to wild-type control levels. Using an *in vitro* incision assay coupled with substrates bearing abasic (Figure 8D and E), 5-OH-Uracil (Figure 8F and G) or 8-oxo-dG (Figure 8H and I) sites, we demonstrated that cells expressing *Rad9* N have higher activity than those with *Rad9*^+^ or *Rad9* C (Figure 8D, F, H). By densitometry quantitation of gel bands from three independent trials and calculating the averages, we found a greater increase in incision activity in *Rad9* N bearing *Rad9^−/−^* cells, relative to those with *Rad9*^+^, or the *Rad9*^+/+^ cells (17% at the abasic site, 33% at 5-OH-Uracil, 27% at 8-oxo-dG substrates; Figure 8E, G, I). Cells producing *Rad9* C showed a much more modest increase in incision activity relative to *Rad9*^−/−^ cells.

## DISCUSSION

Rad9 regulates multiple DNA damage-inducible cell cycle checkpoints and contributes to many DNA repair pathways, including BER, nucleotide excision repair, mismatch repair and homologous recombination repair (20–23). In addition to protein–protein interactions involving HUS1 and RAD1, RAD9 interacts independently with several DNA repair proteins, including RAD51, MLH1, APE1, TDG, OGG1 and NEIL1 (18). In a previous study, we demonstrated that Rad9 deficiency enhances sensitivity of mES cells to DNA damaging agents (30). We show herein that NEIL1 protein levels are reduced in response to RAD9 depletion, and that RAD9-mediated NEIL1 protein level regulation is transcriptional in human prostate cancer cells but post-transcriptional in mES cells. In mouse cells, Rad9 binds to the *Neil1* promoter at a p53-binding consensus sequence, and the same is true for human RAD9 and the *NEIL1* promoter (Figure 3). This is in agreement with our previous observation that RAD9 can bind *p21* promoter sequences (33). We noted a decrease in *NEIL1*-luciferase reporter gene expression either by deleting/mutating the p53-binding sequence in the promoter or by down regulating *RAD9* expression in human prostate cancer cells. This suggests that RAD9 regulates *NEIL1* expression in human cells by binding to the p53 consensus sequences and modulating transcription. It was reported that −900 to +40 of the *NEIL1* promoter is required for CRE/AP-1-mediated NEIL1 activation by oxidative stress (48). Our ChIP results showed that RAD9 binds to the *NEIL1* promoter at −821 to −641, a region that contains two p53-binding sites. Two c/EBP/AP-1 sites, reported to be involved in oxidative stress dependent *NEIL1* activation, are outside of the RAD9 binding region in the *NEIL1* promoter (48). Therefore, RAD9-mediated *NEIL1* regulation is independent of the c/EBP/AP-1 sites. Interestingly, although mouse Rad9 can bind the *Neil1* promoter in mES cells, we did not observe transcriptional regulation. This suggests that perhaps mES cells lack accessory factors or specific chromatin structural attributes near the promoter needed by Rad9 for its role in governing transcription of *Neil1*.

In mES cells, Neil1 protein has a shorter half-life when Rad9 is absent, and our results also show that Neil1 stability is proteasome dependent (Figure 2D). Recently, it was reported that ubiquitin-specific peptidase 20 regulates the clamp loader protein, Rad17 (49). Our results indicate that Neil1 might be regulated in a similar fashion in mES cells.

NEIL1 glycosylase is the first enzyme that acts specifically in short-patch BER, and not only detects lesions but also removes them by intrinsic lyase activity, unlike its mono-functional, related family member NEIL3 (13). NEIL1, but not NEIL2 or NEIL3, has a broad range of target substrates, including 8-oxo-dG, an apurinic site, and 5-OH-Uracil, and is involved in BER as well as nucleotide excision repair (43,48,50). Our data indicate that Rad9-deficient cells have reduced levels of Neil1 protein, and as a result are more sensitive to certain DNA damaging agents. Ectopic expression of *Neil1* or *Rad9* in the Rad9-deficient cells restores the ability to repair damaged DNA. Furthermore, an *in vitro* assay demonstrated that *Rad9*^−/−^ cells have less incision activity, compared to *Rad9*^+/+^ cells. Thus, we demonstrate that Rad9 participates in BER by regulating Neil1 protein level. This finding is consistent with a previous report showing involvement of RAD9 in nucleotide excision repair, by influencing DDB2 protein stabilization (21).

PCNA and RAD9 physically interact with NEIL1, and this stimulates NEIL1 activity (29,51). Our IP results showed that the N-terminal region of Rad9, containing two PCNA-like domains, is responsible for the Rad9–Neil1 interaction. It has been reported that the 9-1-1 complex acts as a DNA damage specific substitute for PCNA, as they have similar structural attributes (20,52). Therefore, it is possible that Rad9, PCNA and Neil1 form a complex. Our results herein show that Rad9 enhances Neil1 protein stability. Cells expressing *Rad9* C, which does not interact with Neil1, have reduced Neil1 protein levels. Cells expressing *Rad9* N are more resistant to DNA damaging agents and have higher incision activity, compared to those containing *Rad9* C. In addition, we observed higher incision activity in cells expressing *Rad9* N, compared to *Rad9*^+/+^ cells and even *Rad9*^−/−^ cells containing *Rad9*^+^. We did not observe any increase in Neil1 protein level in these cells, compared to those bearing *Rad9*^+^. Therefore, the higher incision activity might be due to a Neil1 independent function, or the absence of the C-terminal region of Rad9 might cause hyperactivation of Neil1.

In summary, we demonstrate that RAD9 can regulate *NEIL1* at the transcriptional level in human prostate cancer cells, while in mouse ES cells Rad9 impacts on Neil1 protein levels by controlling proteasomal degradation. We show that these novel regulatory mechanisms determine the sensitivity of mammalian cells to a variety of DNA damaging agents. It is important to define these mechanisms in more detail, and how they coordinate with other functions of RAD9 in cell cycle checkpoint control and apoptosis, as the cellular response to DNA damage is critical for determining whether carcinogenesis or other deleterious events will ensue.

## SUPPLEMENTARY DATA

Supplementary Data are available at NAR Online.

SUPPLEMENTARY DATA
