# Supplementary material for: Regulation of NEIL1 protein abundance by RAD9 is important for efficient base excision repair
Source: Nucleic Acids Res. 2015 Apr 14;43(9):4531–46. doi: 10.1093/nar/gkv327 (PMC4482081; doi:10.1093/nar/gkv327)
Supplement: SUPPLEMENTARY DATA [file supp_43_9_4531__index.html]

Regulation of NEIL1 protein abundance by RAD9 is important for efficient base excision repair — Regulation of NEIL1 protein abundance by RAD9 is important for efficient base excision repair — SUPPLEMENTARY DATA 

# Regulation of NEIL1 protein abundance by RAD9 is important for efficient base excision repair

## SUPPLEMENTARY DATA

**Files in this Data Supplement:**

- SUPPLEMENTARY DATA
- SUPPLEMENTARY DATA
- SUPPLEMENTARY DATA
- SUPPLEMENTARY DATA
- SUPPLEMENTARY DATA
- SUPPLEMENTARY DATA
- SUPPLEMENTARY DATA
