# Supplementary figures and images for: Regulation of NEIL1 protein abundance by RAD9 is important for efficient base excision repair
Source: Nucleic Acids Res. 2015 Apr 14;43(9):4531–46. doi: 10.1093/nar/gkv327 (PMC4482081; doi:10.1093/nar/gkv327)

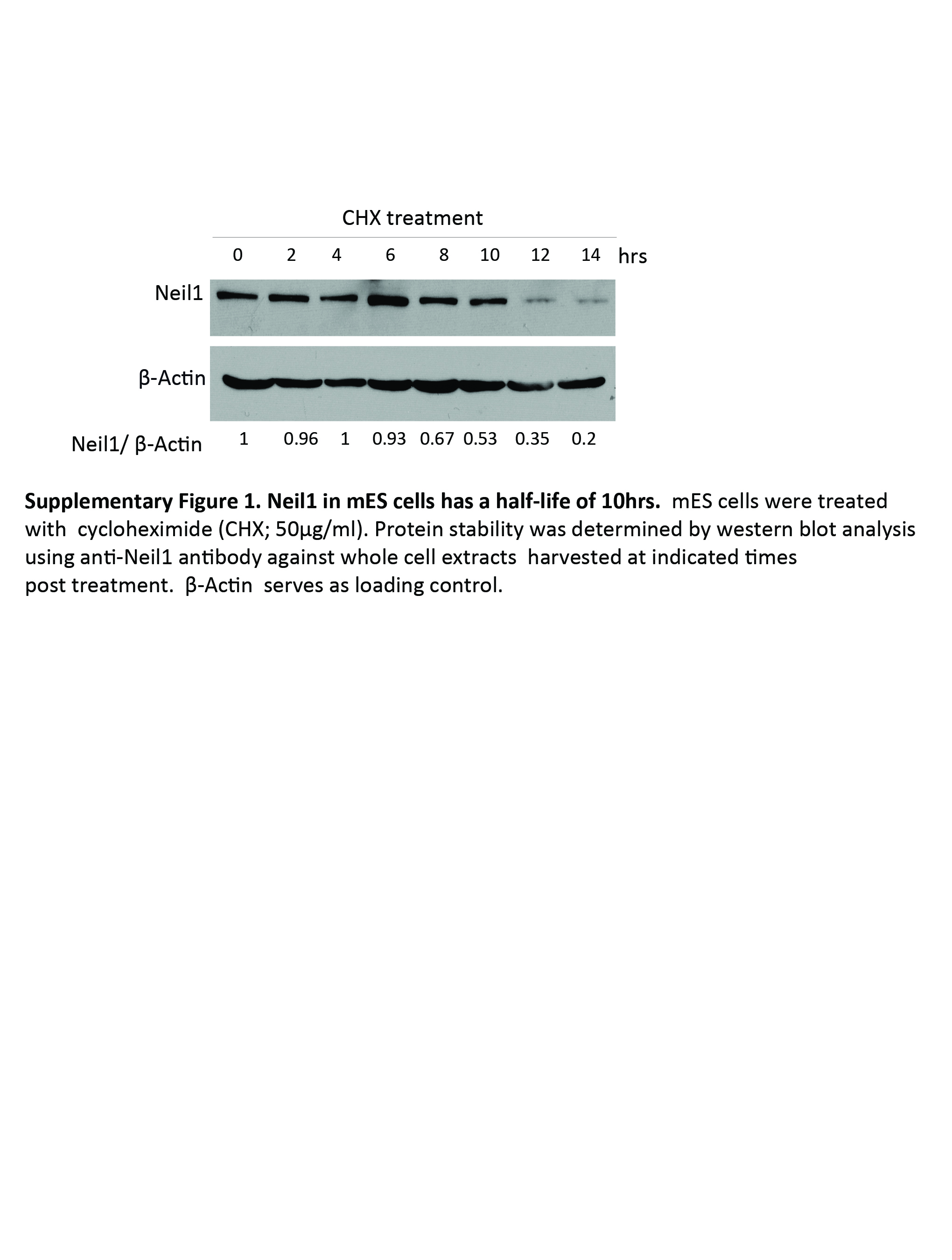

Supplement: SUPPLEMENTARY DATA [file supp_gkv327_nar-03555-d-2014-File010.jpg]

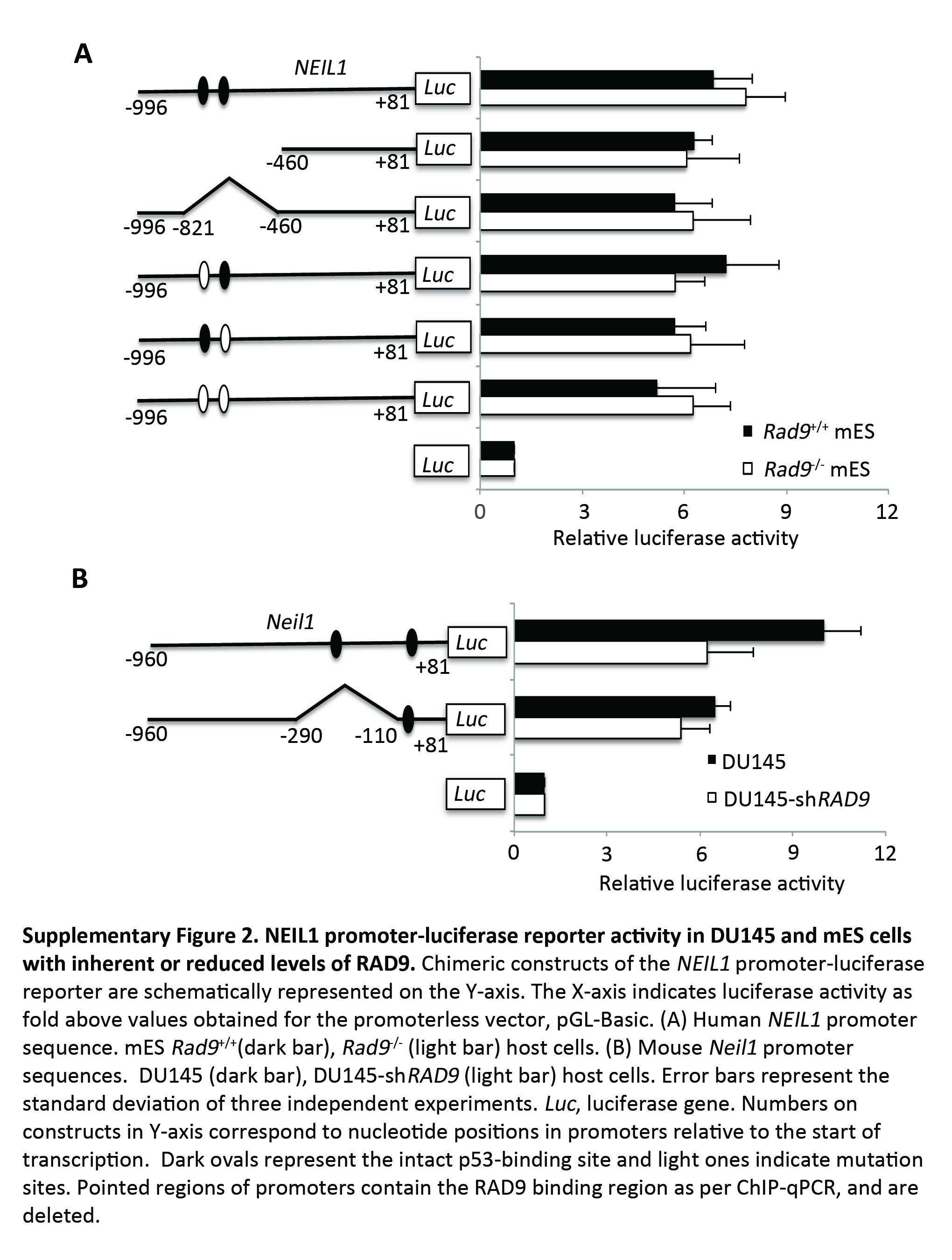

Supplement: SUPPLEMENTARY DATA [file supp_gkv327_nar-03555-d-2014-File011.jpg]

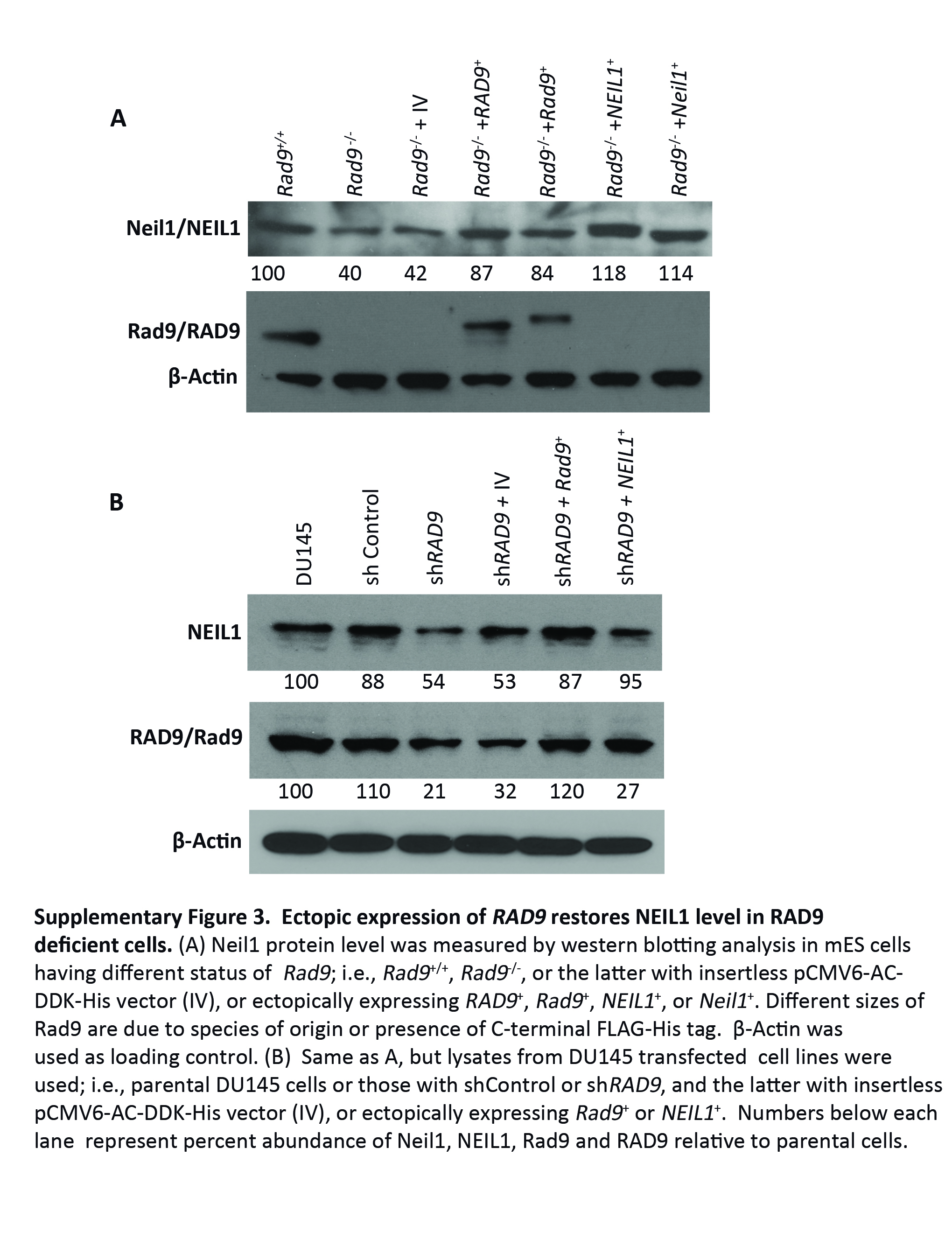

Supplement: SUPPLEMENTARY DATA [file supp_gkv327_nar-03555-d-2014-File012.jpg]

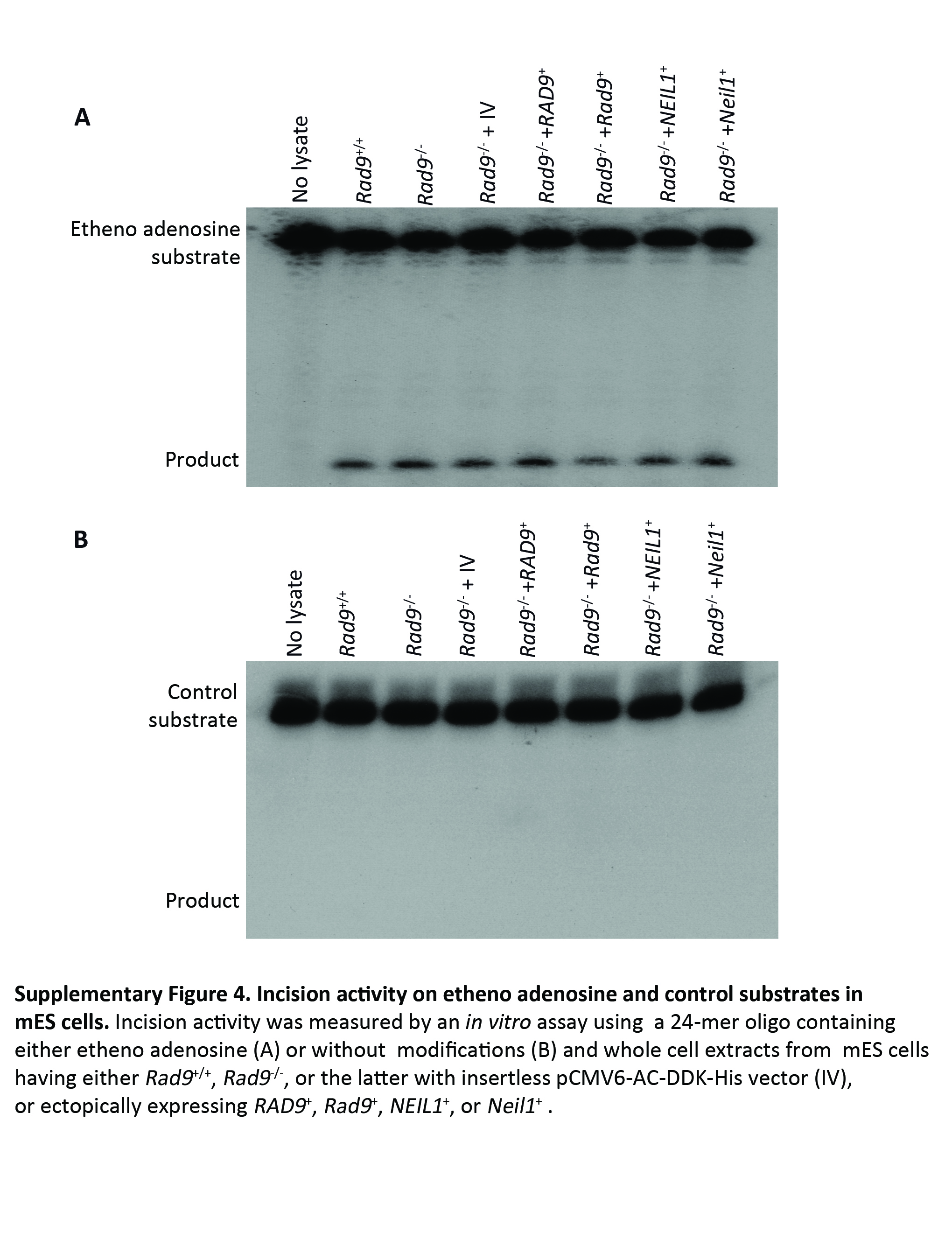

Supplement: SUPPLEMENTARY DATA [file supp_gkv327_nar-03555-d-2014-File013.jpg]

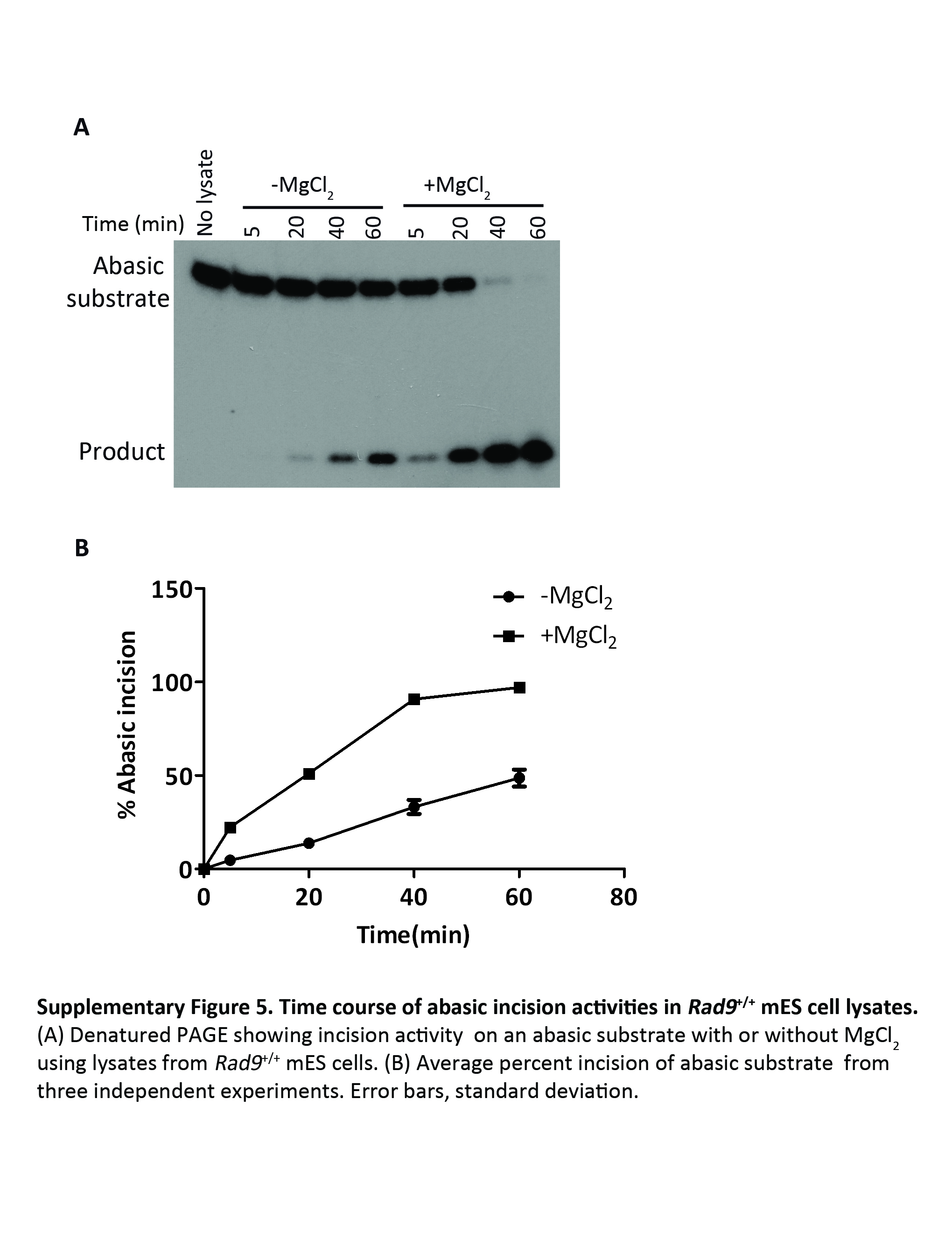

Supplement: SUPPLEMENTARY DATA [file supp_gkv327_nar-03555-d-2014-File014.jpg]

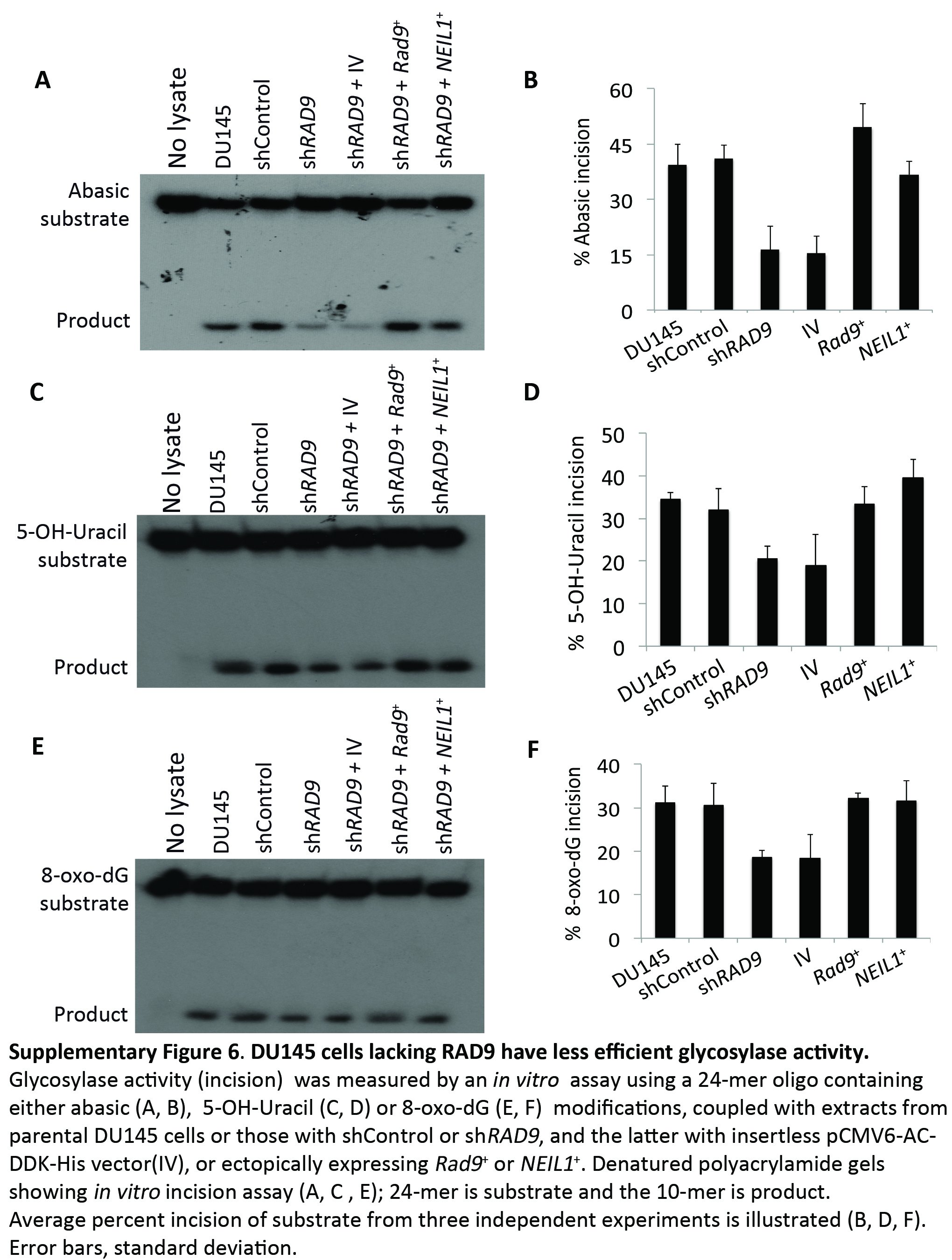

Supplement: SUPPLEMENTARY DATA [file supp_gkv327_nar-03555-d-2014-File015.jpg]
