# Supplementary material for: Regulation of NEIL1 protein abundance by RAD9 is important for efficient base excision repair
Source: Nucleic Acids Res. 2015 Apr 14;43(9):4531–46. doi: 10.1093/nar/gkv327 (PMC4482081; doi:10.1093/nar/gkv327)
Supplement: SUPPLEMENTARY DATA [file supp_gkv327_nar-03555-d-2014-File016.docx]

| **Species** | **Primer name** | **Direction** | **Position** | **Sequence (5’-3’)** |
| --- | --- | --- | --- | --- |
| **Human** | A | Forward | -996 | CGCCTGTAATCCCAACACTT |
|  | A* | Reverse | -821 | AAGTGATCCTCAGCCTCACCCT |
|  | B | Forward | -821 | TGAGCCCGGGAGGTCGGAGGCT |
|  | B* | Reverse | -641 | TTACAGAAGTGAGCCACCATG |
|  | C | Forward | -641 | AATCCCAGCACTTTGGGAGGCC |
|  | C* | Reverse | -460 | GGGTTCAAGTGATTCTCCTGCCT |
|  | D | Forward | -460 | GGGAAGTGGAGGCTGCAGTGAG |
|  | D* | Reverse | -281 | TACTTCTGCCATAATAAATGAA |
|  | E | Forward | -281 | TTTTAAAGTAAATTAGACAAATCA |
|  | E* | Reverse | -101 | GCACTTCATTCTAAGTGTATA |
|  | F | Forward | -101 | AGACTCCACACGGAGGCCCTCC |
|  | F* | Reverse | +81 | GGCGGAAGGAACCGCCAGTACA |
| **Mouse** | A | Forward | -980 | CCCGGGAAAGACAGAGAAACCA |
|  | A* | Reverse | -821 | GAAGGTAGGTAGGGAAGACGGG |
|  | B | Forward | -821 | CTCCTCAGATCTTGCTAGTAGC |
|  | B* | Reverse | -641 | CTGCCTTCTTGTCAGGACAGGA |
|  | C | Forward | -641 | GCCTCTCTCTTCTGCCTGGGTG |
|  | C* | Reverse | -465 | AGCATCTGTTCTAGCACCTCAC |
|  | D | Forward | -465 | GAGTAGCTTTGAACTTTGGGAGC |
|  | D* | Reverse | -290 | CCTATTACCTTGTGTCCCCCAAT |
|  | E | Forward | -290 | AGGCTCCAGCAAGAATCTGAAC |
|  | E* | Reverse | -100 | AGACACAGAGAGGCATAGCGCC |
|  | F | Forward | -65 | ACAAGACATTTCTGGCACGACC |
|  | F* | Reverse | +81 | CACACACCCACCAAATACCAGC |

**Supplementary Table: List of primers used in the ChIP-qPCR studies.** The number in each primer represents their start position with respect to the transcription start site. All reverse primers were marked as *.
